# Student preferences for microbiology laboratory teaching approaches in a problem-based learning curriculum

**DOI:** 10.1099/acmi.0.001180.v3

**Published:** 2026-04-15

**Authors:** Ronni Mol Joji, Archana Prabu Kumar, Amer Almarabheh, Abdulrahman Yusuf Ismaeel, Khalid Bindayna, Eman Farid, Mohd Shadab, Ali Al Mahmeed, Mohammad Shahid

**Affiliations:** 1 Department of Microbiology, Immunology, and Infectious Diseases, College of Medicine and Health Sciences, Arabian Gulf University, Manama, Bahrain; 2 Department of Medical Education, College of Medicine and Health Sciences, Arabian Gulf University, Manama, Bahrain; 3 Department of Family and Community Medicine, College of Medicine and Health Sciences, Arabian Gulf University, Manama, Bahrain

**Keywords:** curriculum, lab preferences, learning, medical students, microbiology

## Abstract

**Background.** Microbiology is a mandatory component in all medical schools. The microbiology lab sessions at Arabian Gulf University focus on students’ ability to demonstrate pre-identified laboratory competencies and relate laboratory data with clinical scenarios. We conducted a questionnaire-based survey among undergraduates to know the preferences with respect to delivery methods for optimally facilitating learning for each laboratory session.

**Methods.** The participants were year 3 and 4 medical students who were engaged in microbiology laboratory instruction as part of their undergraduate medical education. All the participants were asked to complete the structured and validated survey (Google Forms). Descriptive statistics were used to examine the demographic characteristics of the participants and their responses to the scale items.

**Results.** Among 168 student responses, the majority preferred a blended mode of learning of lab sessions across all the 3 years. The preferences appeared to align with the lab objectives. Students preferred theoretical aspects, and case discussions could be delivered online while the skill-based sessions may be conducted face-to-face.

**Conclusion.** The overall trend indicated that students valued the flexibility of blended learning while recognizing the importance of in-person sessions for achieving laboratory competencies and clinical application skills. These findings will support future research in evaluating various delivery methods and contribute to the advancement of high-quality microbiology education.

## Data Summary

All data sets supporting the findings are available in the manuscript.

## Introduction

Education refers to the process of studying and acquiring knowledge. Instruction, teaching, discussion and research are all part of education [1]. An established and widely adopted active instructional approach, problem-based learning (PBL), was first used to teach medicine in the 1950s [2]. To support learners, PBL was introduced since the beginning of the MD programme at Arabian Gulf University (AGU) (1980) [3]. At the institution, the foundational principles and evolving practice of medicine are conveyed through a curated series of case-based pedagogical challenges. These problems are structured to develop clinical acumen while also cultivating an awareness of both community and planetary health priorities. Local and global healthcare landscapes additionally help contextualize learning experiences [4]. Modern physicians must have a basic understanding of microbiology [5] . Clinical microbiology is a mandatory component in all medical schools [6].

The MD programme at AGU is organized into three phases. In phase I, students complete 1 year of basic sciences. In phase II, the curriculum is organized into nine integrated organ- and system-based units delivered over 3 years. PBL serves as the primary educational strategy, with strong emphasis on both its content and learning process. In the final phase III, students undertake supervised clinical training in affiliated hospitals and primary healthcare centres, gaining practical experience in real healthcare settings [7]. The lab skills in microbiology are curated to complement the educational objectives of the PBL curriculum .

The sessions are delivered as wet or dry labs (skills training and theoretical case discussions, respectively) based on the PBL scenarios. The microbiology laboratory sessions provided to these small groups (cohort of 20–25) span a duration of ~2 to 3 h. Learners seek to exhibit pre-defined technical lab skills, discern and contrast prevalent pathogenic microbes, appraise and make sense of diagnostic data and contextualize laboratory findings within relevant clinical presentations [8]. To accomplish these objectives, a series of problems and experimental studies are conducted to guide students through the entire set of lab skills. In the wet labs, all the students are allowed to execute a specific lab skill under the lab instructor’s supervision. In the dry labs, case discussions and theoretical aspects related to the cases are discussed with students. Faculty guide students’ hands-on observation and participation, supplemented by video demonstrations and interactive case scenarios. Throughout, an on-site laboratory instructor facilitates knowledge construction through these varied pedagogical activities and exercises. To contend with growing pressures on physical learning facilities and material resources, there are other institutions that have begun experimenting with calibrating the quantity and modality of laboratory sessions. Specifically, adjustments are being tested such as reducing the number of in-person microbiology lab meetings while supplementing the curriculum with virtual and online complementary components [9].

Prior to implementing substantive reforms to the approach and structure of microbiology instruction, we recognized the importance of obtaining insights from students at AGU. To develop comprehension of the prevailing modality for microbiology laboratory education, surveys were administered to gather input from key stakeholders – namely, third-year and fourth-year undergraduates. The aim of the study was to gain insight into the participants' preferred mode of conducting each of the microbiology lab sessions as the topics and learning outcomes of each lab session differed across the years.

## Methods

### Study participants

The participants in this study were undergraduate medical students in their third and fourth years. We selected third- and fourth-year students as they experienced different methods of teaching (Files S2 and S3 available in the online Supplementary Material).

### Study design

The questionnaires for students were developed based on a thorough literature review and input from experts in the field of microbiology and medical education. The content validation of the questionnaire was done by the Lawshe method by four experts. The consensus of three experts out of four was taken as final approval for each item. Reliability was assessed using appropriate statistics.

All students in their third and fourth years of study were invited to participate in the survey. They were asked to provide their inputs based on their experience of direct exposure to all the teaching modalities (face-to-face, online and blended) across multiple years. The questionnaire consisted of their mode of learning preferences with regard to the learning objectives associated with microbiology labs. For this survey, lab sessions were assessed in three formats: (1) face-to-face, (2) online and (3) blended.

The questionnaire consisted of demographic information and participants’ preferences regarding teaching each lab session across years 2, 3 and 4.

The questionnaire contained the details of each laboratory session with their learning objectives in phase II preclinical phase (years 2, 3 and 4) at AGU. The questionnaire was distributed using Google Forms, and the survey link was shared with the participants through WhatsApp and email.

The study sample consisted of the following:

Year 3: 86 students out of the total of 170 participated in the survey.

Year 4: 82 students out of the total number of 184 participated in the survey.

All study participants (*n*=168) provided informed written consent prior to their involvement in the research.

### Data collection

Data were collected over 2 months from the participants. Participation in the study was completely voluntary. The study did not collect any personal information, and all responses were kept anonymous.

### Data analysis

The responses were collected and subsequently analysed using the Statistical Package for the Social Sciences (SPSS) software, version 28.0 (IBM Corp., Armonk, NY, USA). Following data cleaning and coding, descriptive statistical methods were employed to analyse the participants’ demographic information and scale responses. Frequencies and percentages were computed for the categorical variables.

## Results

### Participants’ demographics

The study included 168 students with an overall mean age of 21.35±2.16 years, and the majority were female (72.6%). Year 3 students had a mean age of 20.93 years, with males averaging 20.96 years and females 20.92 years. In comparison, year 4 students had a higher mean age of 21.78 years, with mean ages of 21.41 years for males and 21.92 years for females.

### Year 2 lab session preferences

Lab sessions conducted in year 2 are given in Table 1. Among the 168 student responses, learning style preferences varied through sessions and year groups. In session 1, 47.7% (*n*=41/86) of year 3 students and 39% (*n*=32/82) of year 4 students expressed a preference for blended learning, reflecting considerable interest in a flexible format. Conversely, 50% (*n*=41/82) of year 4 students opted for face-to-face instruction, highlighting a split preference among the senior cohort.

**Table 1.** Overview of year 2 lab sessions and student preferences

Session (S)	Topic/focus	Technique	Learning outcome	Year 3 (*n*=86)	Year 4 (*n*=82)
S1	Basic microbiology techniques	Staining, culture	Perform and interpret basic microbiology techniques, understand culture techniques and microbial growth characteristics	Blended (47.7%)	Face-to-face (50%)
S2	Immunological reactions	Serological assays – agglutination, immunofluorescence, ELISA and point-of-care tests	Understand and interpret immune responses in the lab, apply immunology principles	Blended (40.7%)	Blended (41.5%)
S3	Bacterial and viral diagnostics	Virus diagnostic tests, culture and identification of *H. influenzae* and *C. diphtheriae*	Identify viral and bacterial pathogens, interpret diagnostic tests accurately	Blended (40.7%)	Blended (46.3%)
S4	Respiratory pathogen detection	Microscopy and culture of sputum specimens for *S. pneumoniae, Legionella, Mycoplasma* and *Chlamydia*	Diagnose common respiratory infections, interpret lab results	Blended (36%)	Blended (47.6%)
S5	Tuberculosis diagnosis	Acid-fast staining, culture, automated and molecular methods	Detect and identify *Mycobacterium tuberculosis*, understand diagnostic techniques	Blended (50%)	Blended (51.2%)
S6	*Streptococci* and rheumatic heart disease serology	Identification of major *Streptococci*, serological tests for rheumatic heart disease	Identify *Streptococcus* species, interpret serology in rheumatic heart disease diagnosis	Face-to-face (44.2%)	Face-to-face (39%) Blended (39%)

**Fig. 1.**
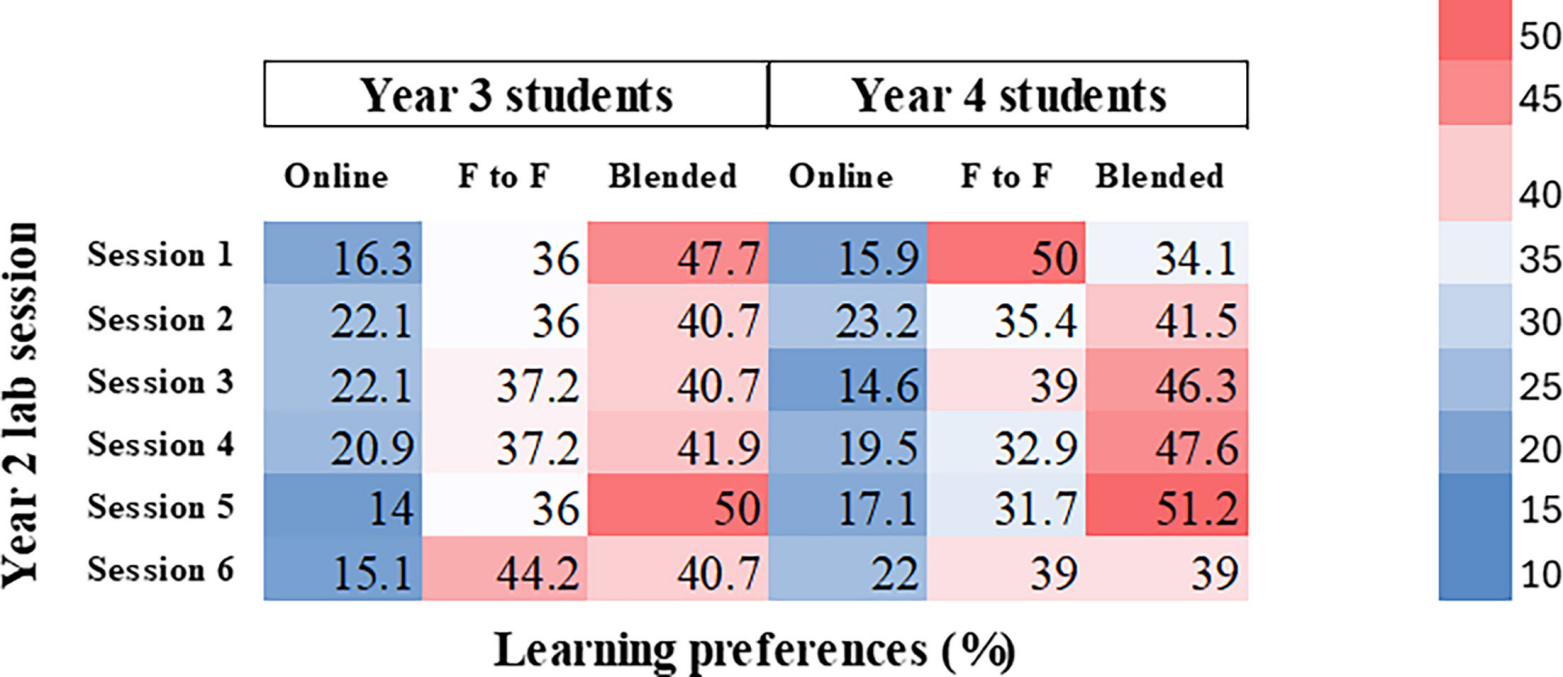
Heatmap illustrating student preferences (per cent) for each year 2 lab session. Each cell (colour block) represents the percentage of students who preferred a particular learning mode for a given session, with darker red shades indicating higher preference percentages and darker blue shades indicating lower preference percentages.

For session 6, 44.2% (*n*=38) of year 3 students and 39% (*n*=32) of year 4 students chose face-to-face mode, emphasizing the need for hands-on experience in practical techniques. Nevertheless, the blended approach remained a common choice for other year 2 lab sessions (Fig. 1), demonstrating an overall inclination towards hybrid learning models.

According to these findings, even while students embrace the flexibility of blended learning, some laboratory sessions, especially those that call for practical technical skills, remain highly appreciated in a conventional, in-person setting. By being aware of these inclinations, curriculum designers may better balance flexibility with hands-on learning.

### Year 3 lab session preferences

Lab sessions conducted in year 3 are given in Table 2. In session 2, a notable 38.4% (*n*=33) of year 3 students preferred online sessions, whereas year 4 students exhibited a strong inclination towards blended learning, with 43.9% (*n*=36) favouring this format.

**Table 2.** Overview of year 3 lab sessions and student preferences

Session (S)	Topic	Technique	Learning outcome	Year 3 (*n*=86)	Year 4 (*n*=82)
S1	Vaginal specimens: *Trichomonas vaginalis* and bacterial vaginosis, *Neisseria gonorrhoeae*	Microscopy, culture, Gram stain, diagnostic criteria for bacterial vaginosis	Identify pathogens, perform wet mount microscopy, interpret lab reports	Blended (45.3%)	Blended (43.9%)
S2	Hepatitis mini cases	Case discussion	Interpret serological profiles, clinical reasoning	Online (38.4%)	Blended (47.6%)
S3	*Helicobacter pylori* infections	Gram stain, culture, serology	Understand the laboratory tests and correlate lab data with symptoms	Blended (43%)	Blended (45.1%)
S4	Stool examination in gastroenteritis	Microscopy for *Entamoeba histolytica, Giardia lamblia*; diagnostic tests for rotavirus, *Campylobacter, Shigella, E. coli* and *Salmonella* species	Identify common GI pathogens and their diagnostic tests, differentiate protozoa vs bacteria vs viruses	Blended (46.5%)	Blended (42.7%)
S5	*Schistosoma* species	Microscopy, egg identification	Identify the stages of *Schistosoma* in samples and their life cycle	Blended (41.9%)	Blended (42.7%)
S6	Urinary tract infections	Urine collection, bacterial count, culture, susceptibility	Interpret urine culture results, choose appropriate therapy based on antibiotic sensitivity test results	Blended (44.2%)	Blended (45.1%)
S7	Nosocomial infections in immunocompromised	Blood collection, blood culture, diagnostic tests for *Candida* species, *Aspergillus* species and *Pseudomonas aeruginosa*	Identify hospital-acquired pathogens, understand sample collection importance, lab tests and interpretation	Blended (54.7%)	Blended (45.1%)
S8	Immunology lab	HLA typing, lymphocyte separation, flow cytometry for markers	Observe immune cell subsets, interpret immunology results	Face-to-face (38.4%)	Blended (45.1%)
S9	Human Immunodeficiency Virus (HIV) diagnosis	Case-based interpretation of serological tests	Apply diagnostic algorithms for Acquired Immune Deficiency Syndrome (AIDS), clinical reasoning	Blended (38.4%)	Blended (41.4%)

In contrast, session 8 demonstrated a different distribution of preferences. While 38.4% (*n*=33) of year 3 students favoured face-to-face learning, 45.1% (*n*=37) of year 4 students continued to show a preference for the blended learning approach.

**Fig. 2.**
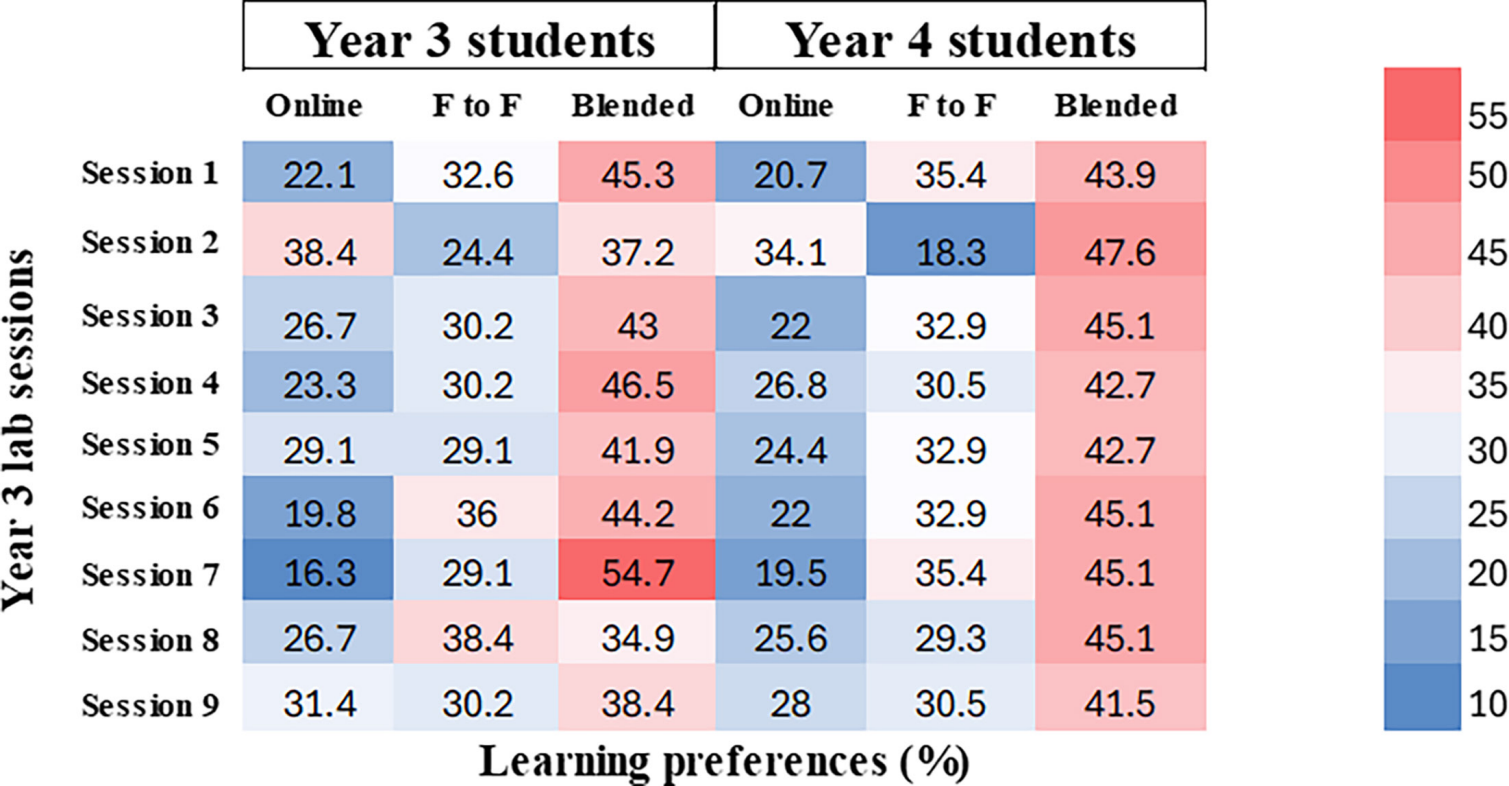
Heatmap illustrating student preferences (per cent) for each year 3 lab session. Each cell (colour block) represents the percentage of students who preferred a particular learning mode for a given session, with darker red shades indicating higher preference percentages and darker blue shades indicating lower preference percentages.

These findings suggest a growing preference for blended learning among senior students, potentially reflecting their need for flexibility alongside hands-on experience. Overall, both third- and fourth-year students demonstrated a clear shift towards blended learning as the preferred mode, reinforcing its effectiveness in modern educational settings (Fig. 2). These insights could inform future curriculum adjustments to better align with student learning preferences and optimize engagement.

### Year 4 lab session preferences

**Fig. 3.**
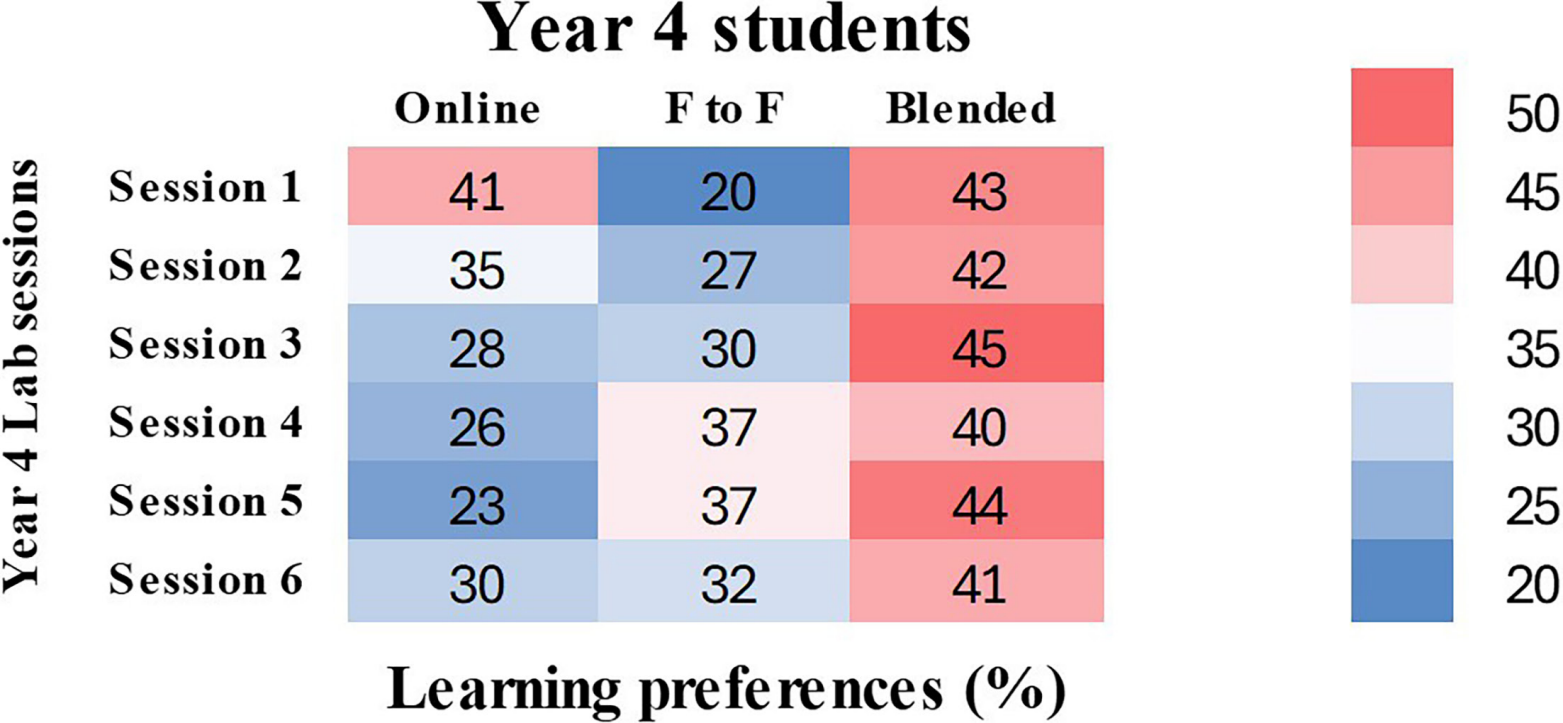
Heatmap illustrating student preferences (per cent) for each year 4 lab session. Each cell (colour block) represents the percentage of students who preferred a particular learning mode for a given session, with darker red shades indicating higher preference percentages and darker blue shades indicating lower preference percentages.

Year 4 lab sessions with objectives are given in Table 3. Across all sessions, blended learning was the most popular mode, with between 40% and 45% of participants choosing it. This suggests that a combination of online materials and real-world, hands-on experience is valued by students. Up to 37% of students favoured face-to-face instruction in classes that required practical laboratory work, such as the microbiological analysis of cerebrospinal fluid (CSF) and the diagnosis of malaria. Overall, preference for online learning was the lowest; the greatest selection rate was 41% for *Staphylococcus* lab diagnosis and the lowest was 23% for malaria diagnosis (Fig. 3). This implies that although students are at ease with distance learning for theoretical subjects, they still favour face-to-face interaction for hands-on activities.

**Table 3.** Overview of year 4 lab sessions and student preferences

Session (S)	Topic	Technique	Learning outcome	Year 4 (*n*=82)
S1	*Staphylococcus* diagnosis	Catalase test, coagulase test, colony characteristics, antibiotic susceptibility testing	Identify *Staphylococcus* species, interpret biochemical tests, assess antibiotic resistance and interpret Methicillin-resistant *Staphylococcus aureus*	Blended (43%)
S2	Rheumatoid arthritis diagnostic tests	Rheumatoid factor, circulating immune complexes, complement activation	Analyse and interpret immunological test results for rheumatoid arthritis	Blended (42%)
S3	Skin ulcers and vector-borne infections	Laboratory tests for skin ulcers, identification of *Leishmania*, venereal Disease Research Laboratory test and Treponema Pallidum Hemagglutination Assay for syphilis	Diagnose skin infections and sexually transmitted infections, interpret serological and microscopic tests for different types of leishmaniasis and different stages of syphilis	Blended (45%)
S4	Central Nervous System (CNS) infections	CSF examination, morphology and staining of *N. meningitidis* and *H. influenzae*, viral cell culture	Identify CNS pathogens, interpret lab findings, understand viral, fungal and bacterial diagnosis based on CSF findings and other lab test reports	Blended (40%)
S5	Malaria diagnosis	Preparation of thick and thin blood films, microscopic identification of malarial parasites	Detect and differentiate *Plasmodium* species and interpret lab tests	Blended (44%)
S6	Post-transplant infections	Case discussion and diagnostic workup	Recognize opportunistic infections, integrate clinical history with lab data	Blended (41%)

CNS, central nervous system.

According to the trends, students favour a hybrid learning style, particularly for intricate microbiological procedures that call for both theoretical understanding and hands-on experience. To optimize student engagement and learning outcomes, future curriculum designs should concentrate on improving blended learning methodologies and combining virtual resources with practical training.

The results of the chi-square test indicated no significant correlation (*P*=0.380) between the preferred learning format for microbiology lab sessions among third- and fourth-year students.

## Discussion

The way knowledge is accessed and transmitted has undergone profound and rapid change because of technology and the digital environment. Learning is no longer an isolated or solo activity, according to Siemens (2005), but has been substituted by something that is done in groups [10]. This study explored the preferences of medical students regarding the mode of delivery for each laboratory session in the microbiology PBL curriculum. Various research has predominantly concentrated on evaluating the effectiveness of different delivery modes of laboratory sessions, placing limited or no emphasis on the perspectives of students involved [11,13]. The responses of students may not be consistent across the board because lab sessions are based on the lab objectives to be met as per the university’s microbiology curriculum considering both the regional and global scenarios.

Year 3 and year 4 students predominantly favoured a blended learning approach for most laboratory sessions in both year 2 (year 3: 5/6 sessions; year 4: 5/6 sessions) and year 3 (year 3: 7/9 sessions; year 4: 9/9 sessions). The preferences are based on the lab objectives, and the mode of delivery depends on the lab content and hands-on experience requirements for the second and third years. The blended learning model preserves essential hands-on laboratory sessions to ensure the acquisition of required practical competencies, while online components will support theoretical understanding and pre-laboratory preparation. Directly handling and seeing specimens instils a sense of responsibility in students, and student collaboration within the laboratory builds respect for the laboratory and builds conceptual understanding [14]. Increased interpersonal connections with instructors and peers are said to boost internal motivation and promote further opportunities to learn through face-to-face communication [15]. Students prefer face-to-face input from instructors over receiving feedback via email, according to research by Salter and Gardner [16].

According to research by Miller *et al.*, there were no statistically significant variations in performance scores between the virtual lab simulation and in-person groups, indicating that both lab modalities are useful for improving learning [13]. Students in that study generally had favourable opinions of virtual lab simulation, citing time management, repetition, thorough explanations and visual learning as its main advantages. Additionally, students expressed interest in using virtual lab simulation as a supplemental lab or as a pre-laboratory activity in a hybrid format. These results validate the use of a hybrid laboratory paradigm to instruct pre-clinical students in a foundational microbiology course [13]. A plethora of studies have shown that the flexibility of online learning is a convenient and cost-effective method of education. Students can easily access labs from anywhere, saving money on lab space and supplies [17]. Furthermore, there are studies that conclude that the online activities allow students to repeat tasks numerous times, a feature that is too costly and time-consuming for face-to-face labs [18].

Despite the benefits of these technologies, none of them can replace hands-on training in a lab [19]. Online sessions must go beyond simply posting lecture notes from a lecturer. While not expanding the educational workload for instructors, good instructional strategy using videos and use of the various available resources in a web-based learning environment can help overcome certain limitations [20]. According to a comprehensive review conducted by Safaeipour *et al.*, virtual laboratories which include fully virtual or remote laboratories as well as laboratory simulations are just as effective as traditional teaching approaches and, in certain situations, even more so.

As a result, virtual laboratories can be a useful substitute or supplementary teaching tool for laboratory sciences [21]. Additionally, giving students practical lab experience helps them grasp the difficulties of experimental microbiology more realistically and guarantees that they are ready for the future [22].

Even in year 4, most of the students (year 4) preferred learning blended style of learning the lab sessions. According to a recent study, learners prefer the pattern of blended learning to a complete face-to-face course, noting differences in the specific instructor, time and location as downsides to face-to-face sessions [23]. Sancho *et al.*, however, emphasized that blended style is distinct to each course [24]. Students prefer only specific features from the two lab delivery designs (face-to-face, online), according to Salter and Gardner’s study of undergraduate viewpoints; however, using online learning as a preparatory aid for other active learning may enhance the students’ perspectives of learning [16]. According to Brockman, owing to the intrinsic fluidity of online learning, some students may find it more suitable for their routines, whereas others may prefer more frameworks. Another reason to use blended learning is the gains that either lab may provide to an expanding and diversified group of students [18]. According to Chitra *et al.*, over 70% of students said the virtual lab was entertaining and they felt safe using it. They believed that the virtual lab offered a fully immersive educational experience. They valued being able to perform each experiment several times without being concerned about errors or accidents. By focusing on the experiments, they could tailor their education as well [25].

All the case discussion sessions were preferred online by students. The key benefit of case discussion is that it connects theoretical principles to clinical circumstances to be solved, helping medical students to better utilize their fundamental science background to solve complicated patient-centred cases. It also helps to enhance and promote hypothesis application, retention and incorporation of learning activities [26]. In a study of medical students using an online interactive case-based discussion format, students reported satisfaction with the learning method and knowledge improvement, and post-test scores were higher than pre-test scores, indicating both perceived benefit and measurable knowledge gain [27].

Giving students the course topic as coursework and then engaging with them for hands-on ‘face-to-face’ learning adds another element to the learning process and reinforces essential themes. If learners do not comprehend what they have read or interacted with in the online component, they will get a second opportunity to listen to a brief review of the subject and participate in a hands-on, real-world lab experience to reaffirm whatever they have studied. Therefore, blended learning is very effective – it strikes the learner from every angle – recite, absorb, perceive and perform [28]. When establishing an undergraduate microbiology course, a great deal of thought and effort is required. However, the time and effort put into this process will be rewarded if it results in a course that gives students with a great learning experience [29].

Nonetheless, our study does have certain limitations that could serve as a foundation for further research in this domain of undergraduate medical training. This research was towards preferred teaching methods for preclinical phase microbiology lab sessions. We included only year 3 and 4 medical students as they had exposure to all the delivery methods (face-to-face, online, blended). Hence, the students’ sample size was small. Since the study involved students only from AGU, the study findings cannot be generalized to other medical schools. Also, the responses were based on self-reported preferences by students; hence, they may not accurately reflect their actual learning outcomes in different delivery modes. Additionally, the study obtained students’ preferences at a single point in time and did not investigate the changes in perception over time. This study was also limited by its reliance on closed-ended questionnaire items, which may not have captured deeper qualitative insights into students’ experiences. Additionally, academic performance outcomes were not evaluated. Furthermore, while blended learning offers flexibility, ensuring consistent student engagement with online components remains a potential challenge, and this aspect was not directly measured in the present study.

## Research implications

The study outcomes can be applied to improve curriculum development at medical schools. Preclinical microbiology lab sessions can be designed with this information in mind, ensuring that they meet the needs and preferences of students within a PBL programme. However, curriculum modifications should not rely solely on student perceptions. Essential competencies requiring hands-on mastery – particularly laboratory techniques, equipment handling and practical skill performance – must be identified by faculty (File S3) in alignment with course learning outcomes, accreditation requirements and professional standards. Improved student engagement and learning outcomes can be attributed to an understanding of preferred teaching and learning approaches. Identifying the preferred teaching method can help the educators, curriculum developers, administrators and policymakers make informed decisions to enhance the teaching and learning experiences in preclinical microbiology lab sessions within a PBL curriculum, ultimately benefiting both faculty and students.

## Conclusion

This study examined undergraduate student preferences for instructional delivery in microbiology labs, focusing on optimizing learning outcomes for pre-clerkship medical students. The results indicated that most students showed a preference for a blended approach to microbiology lab sessions, incorporating both online and face-to-face components. Depending on the microbiology lab objectives in the medical school curriculum, the blended approach might be used in the future. The blended approach can incorporate interactive online activities such as polls, quizzes and breakout room discussions. Theoretical/ pre-lab content can be delivered online, while hands-on practical training and in-person discussions can remain face-to-face to ensure effective skill development. Any curricular modifications will be guided not only by student preferences but also by faculty judgement, alignment with learning outcomes and ongoing evaluation to ensure that educational quality and practical competency development are maintained. This balanced approach will optimize student engagement while preserving the integrity of laboratory-based medical education at AGU. This blended style can also be applied in other fields like pathology and biochemistry where lab skills need to be acquired. Faculty support, clear objectives and well-structured activities could contribute to a positive learning experience and facilitate the acquisition of essential microbiology skills. This study will aid future research to investigate the outcomes of delivering different systems and ensure the development of high-quality microbiology education in medical schools.

## Supplementary material

10.1099/acmi.0.001180.v3 Uncited Supplementary Material 1.

10.1099/acmi.0.001180.v3 Uncited Supplementary Material 2.

10.1099/acmi.0.001180.v3 Uncited Supplementary Material 3.

## Abbreviations

AGU: Arabian Gulf University
PBL: problem-based learning
