# Supplementary material for: Student preferences for microbiology laboratory teaching approaches in a problem-based learning curriculum
Source: Access Microbiol. 2026 Apr 15;8(4):001180.v3. doi: 10.1099/acmi.0.001180.v3 (PMC13082171; doi:10.1099/acmi.0.001180.v3)
Supplement: Uncited Supplementary Material 1. [file acmi-8-01180-s001.pdf]

Student Survey

This survey is to discern students perception for microbiology teaching approaches at Arabian Gulf University.

Please indicate your level of agreement or disagreement with the following statements by ticking one of the answers for each statement.

Possible risks and benefits to the participants: there are no anticipated risks related to participating in this study. You may need to spend 10 to 15 minutes for answering this survey. The possible long term benefit might be the revision of microbiology lab curriculum implementation which could possibly improve student and faculty satisfaction.

Please note that your participation in this study is entirely voluntary and you always have the right to withdraw from the study at any point of time.

This is an anonymous survey. Any information that is obtained during this study will be kept confidential and will be destroyed after the completion of the study.

Ethical considerations:
This study is approved by Research and Ethics committee of Arabian Gulf University.

*Required

1. I give my consent to participate in this survey *

Mark only one oval.

- Yes
- No

2. Gender *

Mark only one oval.

- Male
- Female

3. Age in years *

4. Year of study *

Mark only one oval.

- Year 3
- Year 4

5. In your opinion, what is the preferred method of delivery for each of the following YEAR 2 Microbiology lab sessions *

Mark only one oval per row.

	Online	Face to face	Blended (COMBINATION of online and face to face sessions)
**Session 1-**Basic techniques in microbiology (stain, culture)	⬭	⬭	⬭
**Session 2-**Immunological reactions	⬭	⬭	⬭
**Session 3-**Virus diagnostic tests, Lab diagnosis of Hemophilus species, Lab diagnosis of Corynebacterium diphtheriae	⬭	⬭	⬭
**Session 4-**Microscopical examination and culture of sputum specimen for detection of common respiratory pathogens (S. pneumoniae, Legionella, Mycoplasma, Chlamydia)	⬭	⬭	⬭
**Session 5-**Basic techniques in laboratory diagnosis of TB (acid fast staining, culture, PCR), Interpretation of PPD	⬭	⬭	⬭
**Session 6-** Basic techniques for identification of major Streptococci and serological tests for Rheumatic heart disease	⬭	⬭	⬭

6. In your opinion, what is the preferred method of delivery for each of the following YEAR 3 Microbiology lab sessions *

Mark only one oval per row.

	Online	Face to face	Blended (COMBINATION of online and face to face sessions)
**Session1-**: Preparation and microscopical examination of vaginal secretions for Trichomonas and Bacterial vaginosis, Lab diagnosis of Neisseria gonorrheae	◯	◯	◯
**Session2-**Hepatitis mini cases discussions	◯	◯	◯
**Session3-**Lab diagnosis of Helicobacter pylori infections (Gram stain, culture, serological assays)	◯	◯	◯
**Session4-** Stool examination for identification of Entamoeba histolytica, Entamoeba coli and Giardia, Diagnostic tests for Rotavirus, Campylobacter species, Shigella species, Salmonella species	◯	◯	◯
**Session 5-**Lab diagnosis of Schistosoma species	◯	◯	◯
**Session 6 -**Collection of urine, bacterial count, antibiotic susceptibility, Interpretation of microbiology report of urine culture and sensitivity	◯	◯	◯
	◯	◯	◯

**Session 7**-Laboratory investigations for common agents causing nosocomial infection in the immunocompromised host, procedure of blood collection for culture.			
e**Session 8-**Observe and interpret serological HLA typing plates, T and B lymphocyte separation, Observe lymphocyte surface markers.			
**Session 9-**Interpretation of tests used in the lab diagnosis of HIV infection (mini cases discussion)			

7. Are you currently studying in Year 4? *

Mark only one oval.

- Yes
- No

8. In your opinion, what is the preferred method of delivery for each of the following YEAR 4 Microbiology lab sessions?

Mark only one oval per row.

	Online	Face to face	Blended (COMBINATION of online and face to face sessions)
**Session 1: Lab diagnosis of Staphylococci**	⬭	⬭	⬭
**Session 2- Interpretation of results: Rheumatoid factor, Circulating immune complexes, Complement activation**	⬭	⬭	⬭
**Session 3: Performing lab tests for diagnosing skin ulcers, Identification of Leishmania, Performing and interpreting laboratory tests in diagnosis of syphilis (VDRL, TPHA).**	⬭	⬭	⬭
**Session 4- Examine and interpret microbiological findings of CSF, Demonstration of the morphology, staining characteristics, and culture of N. meningitidis and H. influenzae, Virus- cell culture (mini cases discussion)**	⬭	⬭	⬭
**Session 5: Preparation of thick and thin blood films, Identification of malarial parasites**	⬭	⬭	⬭
**Session 6- Post-transplant microbial infections (case discussion &diagnostic workup)**	⬭	⬭	⬭

This content is neither created nor endorsed by Google.

Google Forms
