# Supplementary material for: Student preferences for microbiology laboratory teaching approaches in a problem-based learning curriculum
Source: Access Microbiol. 2026 Apr 15;8(4):001180.v3. doi: 10.1099/acmi.0.001180.v3 (PMC13082171; doi:10.1099/acmi.0.001180.v3)
Supplement: Uncited Supplementary Material 3. [file acmi-8-01180-s003.pdf]

Supplementary Table 1: Distribution of Laboratory Sessions (MD Program) and modality/modalities used for Year 3 cohort (2021-22)

Year	Unit*	No. of Labs	Mode of Laboratory Delivery (2020-21)	Mode of Laboratory Delivery (2021-22)
Year 2	Unit I	2	Online (Zoom)	F to F#
	Unit II	3	Online (Zoom)	F to F#
	Unit III	1	Online (Zoom)	F to F#
Year 3	Unit IV	1	Online (Zoom)	F to F#
	Unit V	4	Online (Zoom)	Blended
	Unit VI	4	Online (Zoom)	Blended

*In Phase II, the curriculum is organized into nine integrated organ- and system-based units delivered over three years.

F to F: Face to face

Supplementary Table 2: Distribution of Laboratory Sessions (MD Program) and modality/modalities used for Year 4 cohort (2021-22)

Year	Unit*	No. of Labs	Mode of Laboratory Delivery (2019-20)	Mode of Laboratory Delivery (2020-21)	Mode of Laboratory Delivery (2021-22)
Year 2	Unit I	2	F to F#	Online (Zoom)	F to F#
	Unit II	3	F to F#	Online (Zoom)	F to F#
	Unit III	1	F to F#	Online (Zoom)	F to F#
Year 3	Unit IV	1	F to F#	Online (Zoom)	F to F#
	Unit V	4	F to F#	Online (Zoom)	Blended
	Unit VI	4	F to F#	Online (Zoom)	Blended
Year 4	Unit VII	3	F to F#	Online (Zoom)	Blended
	Unit VIII	1	F to F#	Online (Zoom)	Online (Zoom)
	Unit IX	2	F to F#	Online (Zoom)	Online (Zoom)

*In Phase II, the curriculum is organized into nine integrated organ- and system-based units delivered over three years.

F to F: Face to face

		Online	Face to Face	Blended
**Year 2 lab sessions**	**Session 1**	0	43	57
	**Session 2**	14	29	57
	**Session 3**	43	0	57
	**Session 4**	14	43	43
	**Session 5**	29	57	14
	**Session 6**	43	29	29
		**Faculty preferences (%)**		

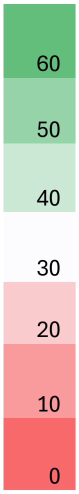

Supplementary Figure 1. Opinion of Faculty on the preferred mode of teaching Year 2 lab sessions

		Online	Face to Face	Blended
**Year 3 lab sessions**	**Session 1**	29	29	43
	**Session 2**	86	0	14
	**Session 3**	29	29	43
	**Session 4**	29	14	57
	**Session 5**	43	14	43
	**Session 6**	0	29	71
	**Session 7**	57	14	29
	**Session 8**	57	0	43
	**Session 9**	86	0	14
		**Faculty preferences (%)**		

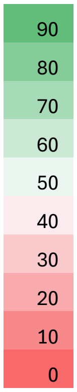

Supplementary Figure 2. Opinion of Faculty on the preferred mode of teaching Year 3 lab sessions

		Online	Face to Face	Blended
Year 4 lab sessions	Session 1	14	14	71
	Session 2	86	0	14
	Session 3	29	29	43
	Session 4	71	0	29
	Session 5	0	29	71
	Session 6	86	0	14
		Faculty preferences (%)		

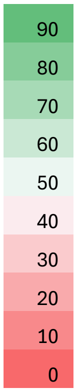

Supplementary Figure 3. Opinion of Faculty on the preferred mode of teaching Year 4 lab sessions
